# Minimally Invasive Oral Rehabilitation Using 3D‐Printed Resins in a Patient With Severe Dental Erosion and Bruxism

**DOI:** 10.1155/crid/4027249

**Published:** 2026-08-17

**Authors:** Gabriela Monteiro, Byron Carpio-Salvatierra, Fabiana Madalozzo Coppla, Fabiana Simas Dreweck, Adriana Postiglione Buhrer Samra

**Affiliations:** ^1^ Department of Dentistry, School of Dentistry, State University of Ponta Grossa, Ponta Grossa, Paraná, Brazil, uepg.br; ^2^ Department of Restorative Dentistry, School of Dentistry, State University of Ponta Grossa, Ponta Grossa, Paraná, Brazil, uepg.br; ^3^ Universidad UTE, Facultad de Ciencias de la Salud Eugenio Espejo, Carrera de Odontología, Quito, Pichincha, Ecuador, ute.edu.ec

**Keywords:** 3D printing, composite resins, computer-aided design, tooth wear, vertical dimension

## Abstract

Oral rehabilitation of patients with severe tooth wear (STW) and loss of vertical dimension of occlusion (VDO) remains a clinical challenge. While ceramics are the traditional choice, 3D‐printed resin composites (3D‐PRCs) have emerged as a minimally invasive and cost‐effective alternative. A young adult patient with severe erosive and attrition wear associated with bruxism presented with an 8‐mm discrepancy between vertical dimension at rest (VDR) and VDO. A fully digital workflow was utilized to plan a 5‐mm VDO increase, maintaining a 3‐mm freeway space. Following clinical validation via a functional mock‐up, the patient was restored using a highly filled 3D‐printed nanohybrid composite (Voxelprint Ceramic; FGM Dental Products, Joinville, SC, Brazil). The additive manufacturing approach allowed for ultraconservative preparations of 24 teeth, guided by a digital wax‐up, prioritizing enamel preservation through minimal surface regularization with chamfer (crowns, onlays, and overlays) and minichamfer (veneers) finish lines. Restorations were cemented using a standardized adhesive protocol: light‐cured resin cement for anterior veneers and dual‐cure resin cement for posterior restorations, including silanization of the 3D‐printed surfaces. According to FDI criteria, the restorations exhibited excellent functional, esthetic, and biological properties, effectively restoring the patient′s vertical dimension and occlusal balance. After that, the patient was referred for the fabrication of a 3D‐milled Michigan occlusal splint. The integration of 3D‐PRCs into a digital workflow provided a predictable and accessible solution for complex functional rehabilitation. This material′s mechanical properties, such as high microhardness or flexural resistance, make it particularly suitable for patients with parafunctional habits. Although short‐term outcomes are promising, longitudinal studies are essential to validate the long‐term performance of ceramic‐reinforced resins as a definitive restorative treatment.

## 1. Introduction

Worn dentition is a widespread condition, affecting approximately 40% of the global population [1]. Due to its multifactorial etiology—including erosion, abrasion, attrition, and bruxism—a rational diagnosis is essential to address both the underlying causes and their clinical sequelae [1, 2]. Beyond managing these factors, oral rehabilitation must restore homeostasis to the stomatognathic system, addressing common complications such as decreased masticatory efficiency, hypersensitivity, and loss of vertical dimension (VD) [3, 4]. In this context, establishing the correct VD is crucial, as traditional methods for determining the vertical dimension of occlusion (VDO) may not be suitable for all worn dentition cases [5].

There are many possible approaches for the treatment of severe tooth wear (STW), including classical full crown preparations. However, scientific and technological advances have guided us toward a new paradigm of minimally invasive dentistry through additive rehabilitations and conservative preparations [6]. It has been well described that tooth preservation is essential to maintain the balance between biological, mechanical, functional, and esthetic parameters [7].

However, material selection for oral rehabilitation remains one of the most controversial topics in contemporary dentistry [8]. Although ceramics are often preferred for their high flexural strength and surface smoothness, composite resins have emerged as a cost‐effective and less invasive alternative [9, 10] due to their more accessible treatment plan without compromising the principles of tooth structure preservation [11], especially in complex rehabilitations where financial constraints may limit the use of ceramic systems. Historically, these resins have been processed via CAD/CAM subtractive technology to produce milled thin restorations with high standardization, internal fit precision, and optimal mechanical properties [4]. More recently, however, additive technology has been introduced to produce definitive restorations using 3D‐printed resin composites (3D‐PRCs) [12].

Additive manufacturing offers the capability to fabricate intricate geometries with customized properties, representing a cost‐effective and accessible alternative for complex rehabilitations [13]. However, since their mechanical performance is strongly dependent on processing variables and material composition, their use as definitive restorations requires careful clinical validation [14, 15].

Given the functional and financial constraints of this case, a 3D‐PRC with high ceramic loading (Voxelprint Ceramic; FGM Dental Products, Joinville, SC, Brazil) was proposed as the definitive material. A recent study [14] showed that this resin exhibits high resistance to wear and fracture, even with reduced thickness (1 mm). Furthermore, since the adhesive properties of printed resins are like those of milled ceramic materials [16, 17], their advantages in a minimally invasive approach [9], combined with more accessible production costs [10], render this treatment a viable alternative to meet the specific demands of this case. Therefore, the objective of this report is to present the comprehensive oral rehabilitation of a young adult patient with STW and loss of VD associated with bruxism, utilizing a fully digital workflow and 3D‐PRCs. This case report was prepared following the CARE statement guidelines [18].

## 2. Case Presentation

**Figure 1:**
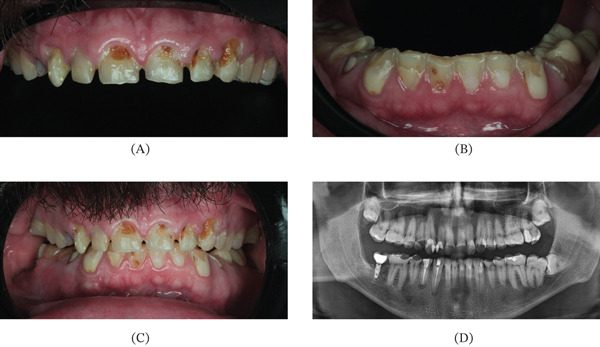
Initial clinical status. (A) Maxillary frontal view. (B) Mandibular frontal view. (C) Intraoral frontal view. (D) Initial panoramic radiograph.

A 34‐year‐old male patient presented to the integrated clinic at State University of Ponta Grossa with main complaints of esthetic dissatisfaction, worn dentition, and masticatory difficulties. Medical history revealed controlled use of antihypertensive, antidepressant, and hypnotic medications. The patient reported daily intake of acidic foods and beverages, as well as a previous diagnosis of bruxism without treatment. Clinical examination revealed generalized STW with features of erosion and attrition (Figure 1A–C); edematous gingiva; cervical carious lesions on Teeth 11, 13, 21, 22, 23, and 41; and a single implant at Position 47. Radiographic findings (Figure 1D) confirmed the necessity for endodontic retreatment in Teeth 44 and 45 due to periapical lesions and defective root canal obturation. Occlusal analysis revealed a Class II molar relationship on the right side and Class I on the left, a deep bite, and loss of VDO (69 mm), with a difference of 8 mm from the vertical dimension at rest (VDR; 77 mm). The proposed treatment was full‐mouth rehabilitation with a 5‐mm increase in VDO using a fully digital workflow.

**Figure 2:**
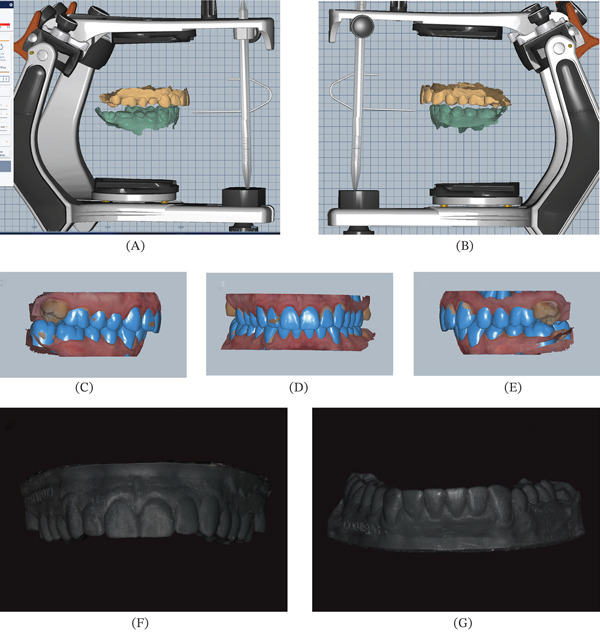
Digital planning. (A, B) Virtual mounting of digital models in a virtual articulator. (C–E) Digital diagnostic wax‐up from lateral and frontal perspectives. (F, G) 3D‐printed physical models generated from the digital wax‐up STL files, used for the fabrication of the functional mock‐up.

**Figure 3:**
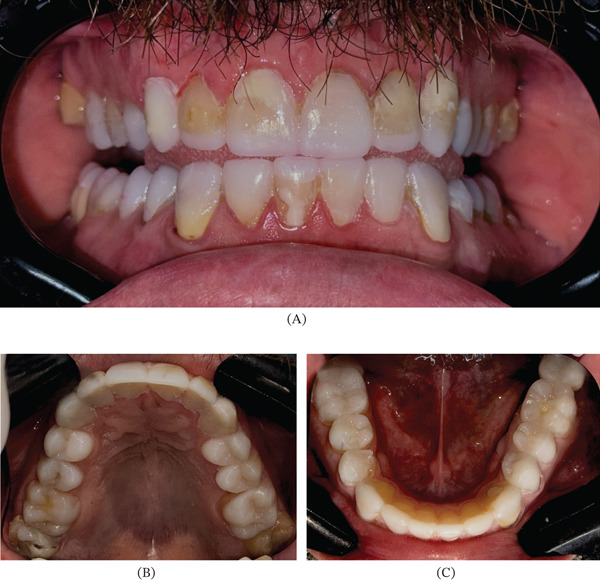
Clinical provisional evaluation. (A) Frontal view of the functional mock‐up used to test the new VDO and neuromuscular adaptation. (B, C) Maxillary and mandibular occlusal views of the anatomical reconstruction. The mock‐up enabled the verification of centric contacts and eccentric guidance before the final restorative phase.

Following the acquisition of the patient′s signed informed consent, an intraoral scan (exocad GmbH, Darmstadt, Germany) was performed to obtain digital models, which were mounted on a virtual articulator (Artex; Amman Girrbach, Koblach, Austria) to re‐establish the VDO with a 4‐mm increase (Figure 2). A digital wax‐up was then used to fabricate an esthetic and functional mock‐up using bis‐acrylic resin (Primma Art; FGM Dental Products, Joinville, SC, Brazil) (Figure 3). After clinical validation of the maxillomandibular relationship (including centric contacts and guidance) and patient approval, carious lesions were restored with composite resin prior to the restorative phase.

**Figure 4:**
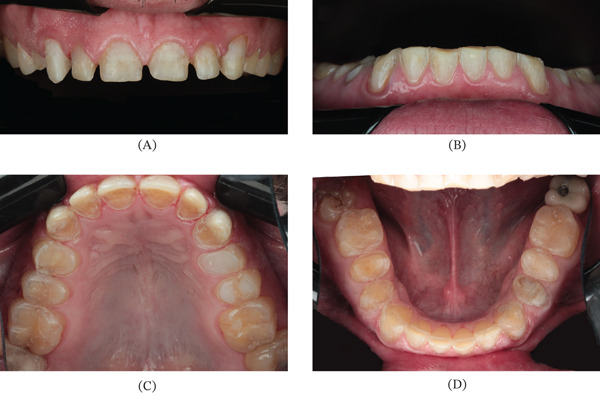
Minimally invasive preparations. (A, B) Frontal views showing conservative reduction and removal of undercuts. (C, D) Maxillary and mandibular occlusal views illustrating enamel preservation.

Minimally invasive tooth preparations were performed, guided by a previously tested additive wax‐up, to prioritize the preservation of enamel and dentin. A 5‐mm increase in the occlusal vertical dimension (OVD) provided sufficient restorative space, allowing for conservative preparation. Diamond burs (#3216 and #3216F; KG Sorensen, São Paulo, Brazil) were used to regularize the occlusal surfaces, maintaining a cusp inclination of approximately 30° for the overlays and onlays. For the buccal and proximal surfaces of the vonlays, a 3°–6° axial inclination was prepared using the same diamond burs. These reductions followed the existing tooth wear patterns to ensure surface regularization and provide a favorable path of insertion. Finish lines were defined as chamfers for the overlays, vonlays, and full‐coverage crowns and minichamfers for the veneers. Preparations for the crowns and veneers provided a minimum restorative space of 0.5 mm, with inclinations following the diagnostic wax‐up design. Palatal surfaces were reduced with #3118 and #3118F diamond burs (KG Sorensen, São Paulo, Brazil) (Figure 4). Preparations were performed on 24 teeth: overlays for molars 16, 26, 36, and 46 and premolars 15 and 25; vonlays for premolars 14, 24, 34, 35, 44, and 45; and full‐coverage crowns for the maxillary anterior teeth (13‐23). Finally, ceramic veneers were placed on the mandibular incisors and canines (33‐43). Provisional restorations were fabricated with autopolymerizing acrylic resin (Dencôr, FGM Dental Products, Joinville, SC, Brazil) and maintained for 8 weeks.

Following this period, once the patient reported comfort and neuromuscular adaptation, a second intraoral scan was performed to design and fabricate the definitive restorations. To maintain the new maxillomandibular relationship (with a VDO of 75 mm and a VDR of 78 mm), transfer guides were fabricated for Teeth 13 and 23. The digital wax‐up STL files served as a reference for the definitive design, ensuring each restoration met the predefined parameters for thickness and occlusal contacts.

The restorations were printed using Voxelprint Ceramic resin (FGM Dental Products, Joinville, SC, Brazil) according to the manufacturer′s instructions, and all restorations were printed at a 90° orientation in a desktop resin printer (Mars 4 DLP; Elegoo, Shenzhen, China). The printed restorations were cleaned with 99.8% isopropyl alcohol and then subjected to postcuring in a light‐curing unit (CyclOne, Elegoo, Shenzhen, China). Characterization of the posterior restorations was performed chairside, beginning with the application of a silane coupling agent (Prosil, FGM Dental Products, Joinville, SC, Brazil) using microbrushes for 60 s, followed by the application of a characterization resin (IPS Empress Direct, Ivoclar Vivadent, Liechtenstein) with a brush and light curing for 20 s at 1200 mW/cm^2^ (Quazar; FGM Dental Products, Joinville, SC, Brazil). A glaze (Angelus, Londrina, PR, Brazil) was then applied and light‐cured for 20 s.

The restorations were cleaned with 70% ethanol and silanized for 60 s. A layer of adhesive (Ambar Universal APS, FGM Dental Products, Joinville, SC, Brazil) was then applied and light‐cured for 20 s. Simultaneously, the tooth structure was cleaned with pumice and etched with 37% phosphoric acid for 30 s, followed by adhesive application. The restorations were then seated using a dual‐cure resin cement (Allcem Dual, FGM Dental Products, Joinville, SC, Brazil), and after excess removal, a final light‐curing was performed for 60 s per surface.

**Figure 5:**
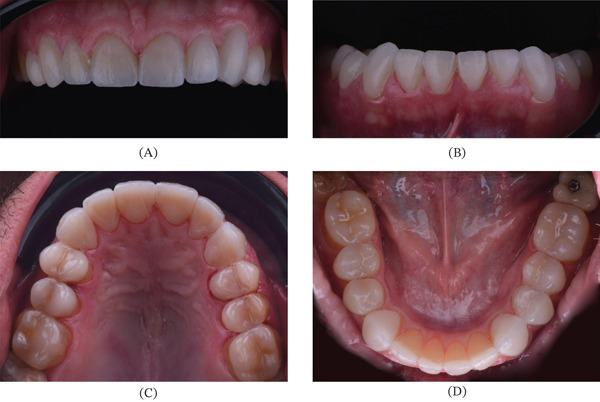
Definitive rehabilitation outcome. (A, B) Maxillary and mandibular frontal views of the 3D‐PRCs showing satisfactory esthetic integration and anatomical recovery. (C, D) Occlusal views displaying the reconstructed morphology.

The anterior restorations were adapted and sent to the laboratory for characterization using shade A1 (VITA Classical; VITA Zahnfabrik, Bad Säckingen, Germany). The cementation protocol was like that described for the posterior restorations; however, an opaque resin cement (Allcem Veneer APS, Opaque White; FGM Dental Products, Joinville, SC, Brazil) was used (Figure 5).

**Figure 6:**
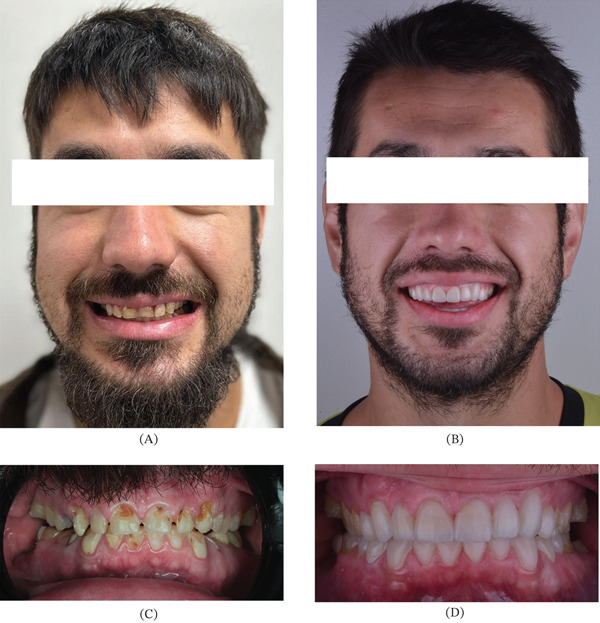
Comparison of initial and final status. (A, C) Preoperative facial and intraoral views showing STW and compromised smile esthetics. (B, D) Postoperative results showing the re‐establishment of the VDO, anatomical recovery, and enhanced facial harmony.

The patient was evaluated 1 week after cementation to establish a baseline, following the FDI criteria across the functional, esthetic, and biological domains [19]. All indirect restorations demonstrated excellent performance in each category (Table S1). This clinical outcome is further illustrated in Figure 6, which provides a comparative view of the patient′s initial and final extraoral appearance. After that, the patient was referred for the fabrication of a 3D‐milled Michigan occlusal splint.

A follow‐up evaluation was conducted 1 month later; however, no differences were observed in the evaluation criteria (Table S1).

## 3. Discussion

The rehabilitation of extensive tooth wear associated with bruxism presents a complex clinical issue, as it requires a combination of procedures within an optimized clinical protocol, including the type of tooth preparation performed and the selected restorative material. Additionally, the ideal maxillomandibular relationship must be carefully determined to ensure successful outcomes. In this clinical case, the optimal position was established by the relationship between the VDR and VDO. An initial 8‐mm difference was identified, allowing for a 5‐mm VDO increase while preserving a 3‐mm freeway space [20]. This planned dimension was verified through facial proportions and landmarks; however, it is important to note that the application of edentulous assessment methods in dentate patients remains a subject of academic debate [5].

The freeway space is not a static measurement, as it varies with mood, head position, and oral behaviors [21]. To ensure accuracy in this case, measurements were standardized and repeated across different sessions, yielding consistent results. A facially driven planning approach was adopted with the clinical intent of respecting the anterior and posterior tooth positions while following literature‐supported principles that favor the temporomandibular joint space and upper airways [5, 22]. This strategy combined traditional and contemporary methods, which were clinically validated through an esthetic and functional mock‐up. The mock‐up allowed for the assessment of occlusal guidance, incisal display, and the occlusal plane (including Spee and Wilson curves). Following the approval of the planned anatomy and maxillomandibular relationship, tooth preparations and provisionalization were initiated to restore the balance of the stomatognathic system.

Adopting a minimally invasive approach is critical in cases of STW to avoid disrupting the biomechanical balance of the remaining structure [23]. In this case, preserving the enamel was prioritized not only to maximize bond strength but also to optimize force dissipation and long‐term restorative performance. Consequently, preparations were limited to smoothing surfaces and removing undercuts, with the 3D planning and mock‐up acting as precise reduction guides. This strategy allowed for a varied design (ranging from onlays to laminates) tailored to the specific wear of each tooth. For the maxillary anterior teeth, although full‐coverage restorations were required due to pre‐existing staining and buccal lesions, the preparations remained ultraconservative by incorporating the patient′s existing incisal and palatal wear into the final design.

Another critical factor is the range of masticatory forces encountered in clinical reality, particularly the multidirectional loads typical of bruxism. In scenarios where a minimum interocclusal space of 1.5 mm cannot be achieved, CAD/CAM composite resins could be considered the material of choice [5, 24]. Although their mechanical properties are lower than ceramic materials [25], CAD/CAM resins exhibit superior fatigue resistance in reduced thicknesses compared to glass–ceramics. This performance is likely attributed to the fact that failure induced by tensile stress is more sensitive to the elastic modulus ratio between the restoration, luting agent, and dentin than to the material′s uniaxial flexural strength. Therefore, the enhanced clinical performance of these restorations is directly related to the elastic compatibility between the composite and dentin [11]. This similarity provides resilient behavior that helps attenuate functional loads on the stomatognathic system, particularly when applied to both arches [14, 26]. Current literature supports the use of composite resins in minimal intervention additive rehabilitations, demonstrating reliable clinical outcomes [27].

However, its performance is highly dependent on processing variables, such as printing orientation [28], layer thickness [29], and postcuring protocols [30], which are still under extensive evaluation. Furthermore, the microstructure of 3D‐PRCs varies significantly among brands, suggesting that clinical success requires a considerable learning curve.

The mechanical behavior of the 3D‐PRC employed in this case is mainly dictated by its monomeric matrix. Indeed, previous studies [13] have demonstrated that high‐molecular‐weight monomers, specifically Bis‐GMA and UDMA, provide the necessary structural integrity for definitive restorations, although their high viscosity necessitates the incorporation of nonhydroxylated monomers like Bis‐EMA and diluents such as TEGDMA to optimize rheology and printability. Unlike milled composites, which benefit from industrialized high‐pressure polymerization and homogeneous filler distribution, 3D‐PRC exhibits layered macrostructures and occasional spherical pores inherent to the additive process [14].

On the other hand, Raman spectroscopy analysis of the specific 3D‐PRC used in this case, as reported by Lozada et al. [14], revealed a balanced monomeric composition of Bis‐GMA, UDMA, and Bis‐EMA. This chemical profile correlates with an optimal degree of conversion and enhanced fracture resistance. Furthermore, the manufacturer′s “intelligent stability system” (ISS) appears to mitigate common additive manufacturing issues by improving rheological stability and color persistence [31]. While the short‐term performance in this patient is promising, longitudinal data remains the definitive requirement to validate these ceramic‐reinforced resins as a long‐term solution for extensive oral rehabilitations.

According to ISO 4049 [32], minimal flexural strength for definitive 3D‐PRC should be ≥ 80 MPa, and hardness should be between 30 and 80 HV. The 3D‐PRC used in this case fits these parameters, presenting a mean of 131 MPa and 42 HV [31]. An independent study [14] reported values of 125.7 ± 9.7 MPa for flexural strength and 34.4 ± 1.1 HV for hardness, concluding that mechanical performance is determined not by a single factor but by the complex interplay among monomer composition, filler content, and photoinitiator efficiency. In general, 3D‐PRCs demonstrate marginal adaptation similar to other well‐established definitive materials [33]. The immediate result was functionally and esthetically pleasant. It was observed that satisfactory color matches between restorations and general patient satisfaction occurred after cementation and during short follow‐up, according to FDI criteria (Table S1).

Despite the favorable esthetic and functional outcomes achieved in this case, certain inherent limitations must be acknowledged. The primary constraint is the short‐term nature of the results. Although current evidence suggests that the mechanical properties of 3D‐PRCs are sufficient for definitive use, longitudinal clinical studies with larger cohorts are imperative to validate their long‐term performance. Furthermore, evidence specifically supporting the efficacy of the resin utilized in this case remains largely dependent on in vitro data. Consequently, a greater volume of clinical research is required to establish standardized protocols and confirm the reliability of these materials for complex oral rehabilitations.

## 4. Conclusion

The adhesive oral rehabilitation using a highly filled nanohybrid 3D‐PRC proved to be a viable and cost‐effective treatment for managing several types of tooth wear. This approach prioritized tooth structure preservation while achieving favorable esthetic and functional integration. The fully digital workflow, complemented by a provisionalization phase, facilitated a successful re‐establishment of the VDO and clinical adaptation, resulting in high patient satisfaction. While short‐term clinical outcomes are promising, longitudinal studies remain essential to validate the long‐term performance of 3D‐PRC materials for definitive and extensive rehabilitations.

## Funding

This study was funded by the Coordenação de Aperfeiçoamento de Pessoal de Nível Superior (10.13039/501100002322) (001) and the Conselho Nacional de Desenvolvimento Científico e Tecnológico (10.13039/501100003593) (304817/2021‐0 and 3008286/2019‐7).

## Ethics Statement

Written informed consent was obtained from the patient for the publication of this case report. As a single case report with explicit patient consent, further ethical review was not required.

## Conflicts of Interest

The authors declare no conflicts of interest.

## Supporting information


**Supporting Information** Additional supporting information can be found online in the Supporting Information section. Table S1: Evaluation of each indirect restoration classified according to the World Dental Federation Criteria (FDI) for functional, esthetic, and biological properties, including patient satisfaction [19].

## Data Availability

The data that support the findings of this study are available in the supporting information of this article.
