# Supplementary material for: Minimally Invasive Oral Rehabilitation Using 3D‐Printed Resins in a Patient With Severe Dental Erosion and Bruxism
Source: Case Rep Dent. 2026 Aug 17;2026:4027249. doi: 10.1155/crid/4027249 (PMC13478791; doi:10.1155/crid/4027249)
Supplement: Supplementary file 1 [file CRID-2026-4027249-s001.docx]

| **FDI criteria (*)** | F1. Fracture of material and retention | | F2. Marginal adaptation | | F3. Contact point/food impact | | F4. Form and contour | | F5. Occlusion and weaR | | B1. Recurrence of caries | | B2. Dental hard tissue defects at the restoration margin | | B2. Postoperative hypersensitivity and pulpal status | | A1. Surface luster and surface texture | | A2. Marginal staining | | A3. Color match | | M1. Patient Satisfaction | | |
| --- | --- | --- | --- | --- | --- | --- | --- | --- | --- | --- | --- | --- | --- | --- | --- | --- | --- | --- | --- | --- | --- | --- | --- | --- | --- |
| **Evaluation time** | Initial | One month | Initial | One month | Initial | One month | Initial | One month | Initial | One month | Initial | One month | Initial | One month | Initial | One month | Initial | One month | Initial | One month | Initial | One month | Initial | One month |  |
| 16 | A | A | A | A | A | A | A | A | A | A | A | A | A | A | A | A | A | A | A | A | C | C | A | A |  |
| 15 | A | A | A | A | A | A | A | A | A | A | A | A | A | A | A | A | A | A | A | A | C | C | A | A |  |
| 14 | A | A | A | A | A | A | A | A | A | A | A | A | A | A | A | A | A | A | A | A | A | A | A | A |  |
| 13 | A | A | A | A | A | A | A | A | A | A | A | A | A | A | A | A | A | A | A | A | A | A | A | A |  |
| 12 | A | A | A | A | A | A | A | A | A | A | A | A | A | A | A | A | A | A | A | A | A | A | A | A |  |
| 11 | A | A | A | A | A | A | A | A | A | A | A | A | A | A | A | A | A | A | A | A | A | A | A | A |  |
| 21 | A | A | A | A | A | A | A | A | A | A | A | A | A | A | A | A | A | A | A | A | A | A | A | A |  |
| 22 | A | A | A | A | A | A | A | A | A | A | A | A | A | A | A | A | A | A | A | A | A | A | A | A |  |
| 23 | A | A | A | A | A | A | A | A | A | A | A | A | A | A | A | A | A | A | A | A | A | A | A | A |  |
| 24 | A | A | A | A | A | A | A | A | A | A | A | A | A | A | A | A | A | A | A | A | A | A | A | A |  |
| 25 | A | A | A | A | A | A | A | A | A | A | A | A | A | A | A | A | A | A | A | A | A | A | A | A |  |
| 26 | A | A | A | A | A | A | A | A | A | A | A | A | A | A | A | A | A | A | A | A | B | B | A | A |  |
| 36 | A | A | A | A | A | A | A | A | A | A | A | A | A | A | A | A | A | A | A | A | B | B | A | A |  |
| 35 | A | A | A | A | A | A | A | A | A | A | A | A | A | A | A | A | A | A | A | A | A | A | A | A |  |
| 34 | A | A | A | A | A | A | A | A | A | A | A | A | A | A | A | A | A | A | A | A | A | A | A | A |  |
| 33 | A | A | A | A | A | A | A | A | A | A | A | A | A | A | A | A | A | A | A | A | A | A | A | A |  |
| 32 | A | A | A | A | A | A | A | A | A | A | A | A | A | A | A | A | A | A | A | A | A | A | A | A |  |
| 31 | A | A | A | A | A | A | A | A | A | A | A | A | A | A | A | A | A | A | A | A | A | A | A | A |  |
| 41 | A | A | A | A | A | A | A | A | A | A | A | A | A | A | A | A | A | A | A | A | A | A | A | A |  |
| 42 | A | A | A | A | A | A | A | A | A | A | A | A | A | A | A | A | A | A | A | A | A | A | A | A |  |
| 43 | A | A | A | A | A | A | A | A | A | A | A | A | A | A | A | A | A | A | A | A | A | A | A | A |  |
| 44 | A | A | A | A | A | A | A | A | A | A | A | A | A | A | A | A | A | A | A | A | A | A | A | A |  |
| 45 | A | A | A | A | A | A | A | A | A | A | A | A | A | A | A | A | A | A | A | A | A | A | A | A |  |
| 46 | A | A | A | A | A | A | A | A | A | A | A | A | A | A | A | A | A | A | A | A | B | B | A | A |  |

(*) A = clinically excellent/very good; B = clinically good; C = clinically satisfactory; D = clinically unsatisfactory and; E = clinically poor.

**Supplementary Table S1.** Evaluation of each indirect restoration classified according to the World Dental Federation Criteria (FDI) for functional, aesthetic and biological properties, including patient satisfaction [19].
